# Structural Examination of Halogen-Bonded Co-Crystals of Tritopic Acceptors

**DOI:** 10.3390/molecules23010163

**Published:** 2018-01-13

**Authors:** Stefan N. L. Andree, Abhijeet S. Sinha, Christer B. Aakeröy

**Affiliations:** Department of Chemistry, Kansas State University, Manhattan, KS 66506, USA; snlandree@ksu.edu (S.N.L.A.); sinha@ksu.edu (A.S.S.)

**Keywords:** halogen bond, σ-hole, tritopic acceptor, co-crystals, crystal structures

## Abstract

A series of tritopic *N*-heterocyclic compounds containing electrostatically and geometrically equivalent binding sites were synthesized and subjected to systematic co-crystallizations with selected perfluoroiodoarenes in order to map out their structural landscapes. More than 70% of the attempted reactions produced a co-crystal as indicated by IR spectroscopy. Four new crystal structures are reported and in all of them, at least one potential binding site on the acceptor is left vacant. The absence of halogen bonds to all sites can be ascribed primarily due to deactivation of the σ-hole on the iodo-arene donors and partially due to steric hindrance. The tritopic acceptors containing 5,6-dimethylbenzimidazole derivatives yield discrete tetrameric aggregates in the solid state, whereas the pyrazole and imidazole analogues assemble into halogen-bonded 1-D chains.

## 1. Introduction

The anisotropic charge density around polarizable halogen atoms has produced considerable interest in halogenated compounds in the context of noncovalent interactions [1,2,3,4,5]. The halogen bond (XB) has been thoroughly investigated in the crystalline solid-state by Resnati [6,7], Hanks [8] and others [9,10], and according to the 2013 official definition, a halogen bond “occurs when there is evidence of a net attractive interaction between an electrophilic region associated with a halogen atom in a molecular entity and a nucleophilic region in another, or the same, molecular entity” [11]. Important features of the XB include directionality [12,13,14], strength [15,16,17,18], polarizability [19], tunability [5,20,21], hydrophobicity [22] and donor-atom dimension [9,22]. The rapid growth of successful applications of XB in fields such as drug development [23] and materials design [23,24] has contributed to the popularity of this field.

The ability of activated halogen atoms to interact with neutral or ionic Lewis bases (halogen-bond acceptors) has received considerable attention in practical crystal engineering [25] but numerous issues need to be resolved before halogen-bond-based synthetic strategies attain the reliability and versatility that we associate with covalent synthetic transformations. It is not yet clear how we can control chemical compositions and stoichiometries of targeted products when attempting to synthesize co-crystals of reactants that carry multiple donor- and acceptor sites. 

**Scheme 1:**
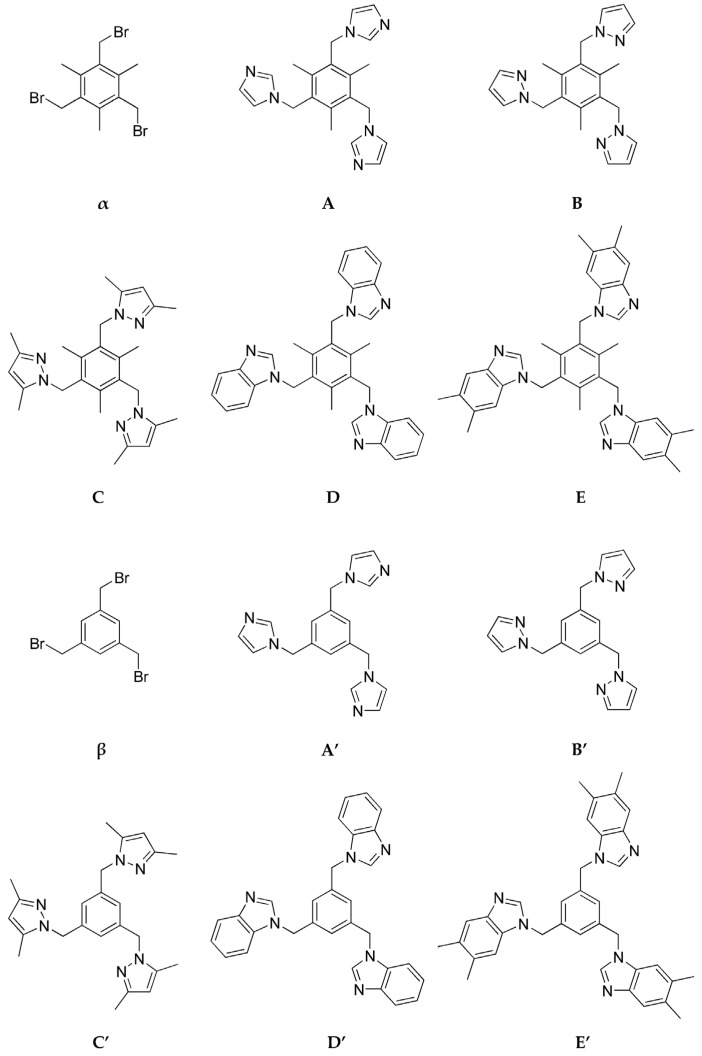
Tritopic precursors and acceptors used in this study.

With this in mind, we opted to examine the structural landscape surrounding a series of tritopic *N*-heterocyclic compounds capable of accepting three halogen bonds. The *N*-acceptor atoms reside on an imidazole, pyrazole or benzimidazole site, respectively, Scheme 1. All the acceptors are conformationally flexible with three geometrically and electrostatically equivalent binding sites.

**Scheme 2:**
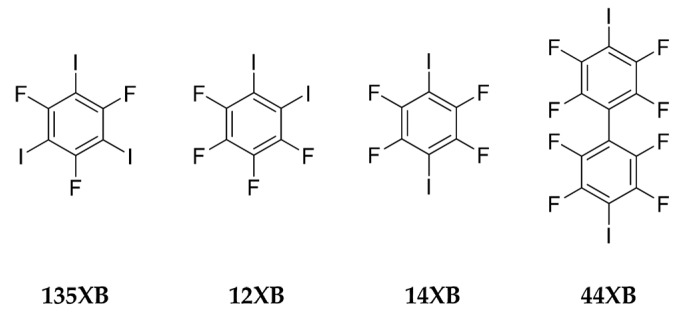
Halogen bond donor molecules used in this study.

The four halogen-bond donors, **14XB, 12XB, 135XB,** and **44XB**, Scheme 2, contain geometrically equivalent binding sites and are very rigid, unlike the tritopic acceptors. In terms of electrostatic surface potentials, all sites are equivalent, prior to forming a halogen-bond, but as has been recognized by van der Boom [26] and Formigue et al. [27], sequential deactivation may take place at the remaining sites upon binding. Since the halogen atoms are substantially larger than their hydrogen-bond donor counterparts [12], additional steric hindrance may prevent interactions from taking place in the intended manner [22]. A study conducted by Schollhorn and co-workers [9] using 1,4-diiodotetrafluorobenzene (**14XB**) with 4,4′-,2,2′- and 2,4′-bipyridine shows that steric consideration are responsible for switching the supramolecular networks from infinite 1-D chains to well-defined ter-molecular complexes. In an attempt to determine enthalpy−entropy compensation of DNA Holliday junctions containing halogenated uracil bases [28], it was found that the most stable pairing involved a bromine halogen-bond donor because the inherent polarizability advantage of the iodine halogen-bond donor was compensated for by the steric disadvantage resulting from its greater size.

Herein we report the synthesis of four co-crystals containing rigid perfluoroiodoarenes and flexible tritopic ligands using halogen-bond interactions as a vector for driving the co-crystal synthesis. With the use of common concepts that define the nature of halogen bonding, we wanted to rationalize the structural outcomes against a backdrop of geometric data from the experimental crystal structure determinations and calculated molecular electrostatic potential surfaces (MEPs).

## 2. Results

Molecular electrostatic potential calculations were performed on the halogen bond donors as means of ranking the expected capability of each donor site, Table 1. With an initial co-crystal screening; Table 2, four crystals suitable for single crystal X-ray diffraction were obtained; **14XB:E, 135XB:E, 12XB:B, 135XB:A**. A summary of the crystallographic data is included in Table 3, and halogen-bond geometries are listed in Table 4.

### 2.1. Description of Solid State Architectures

**Figure 1:**
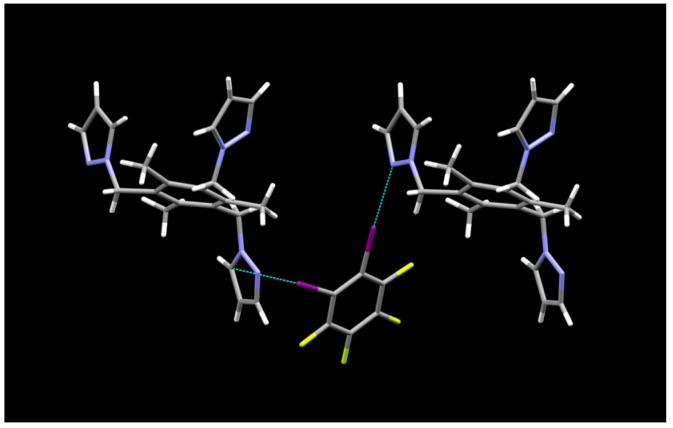
Main interactions in the crystal structure of **12XB:B**.

The attempted co-crystallization of **12XB** and **B** resulted in the formation of a 1:1 binary solid, Figure 1. The primary halogen bond in the structure of **12XB:B** takes place between one of the iodine atoms and N(pyz) forming a C—I⋯N conventional halogen bond and the second iodine atom engages in a C—I⋯π (pyz) interaction with an adjacent pyrazole moiety. These two interactions result in 1D infinite chains. Two remaining pyrazole acceptor sites do not form interactions via halogen bonds but rather form identical two-point short contacts with a methyl C–H on the benzene scaffold of another overlapping tritopic acceptor.

**Figure 2:**
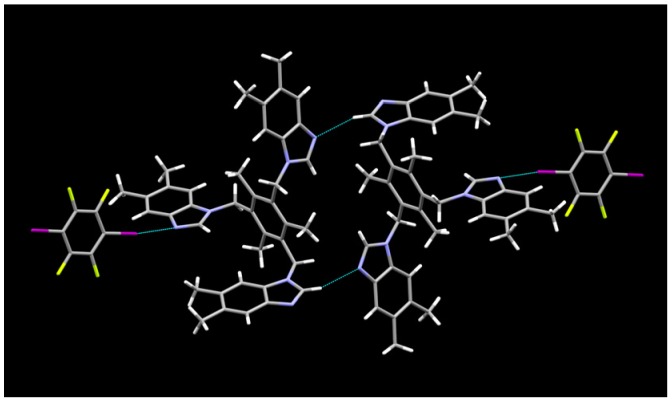
Tetramer in the structure of co-crystal **14XB:E**.

The primary feature in the 1:1 co-crystal **14XB:E** is a discrete tetramer built around a centrosymmetric (central) (bzim)C–H⋯N/N⋯H–C(bzim) hydrogen bonded homo-synthon, which is then extended via two symmetry-related C–I⋯N(bzim) halogen bonds, Figure 2. Only one of the two iodine atoms in **14XB** participates in a halogen bond. The remaining heterocyclic nitrogen atom on the tritopic acceptor forms a short contact with an aromatic C–H moiety of a neighboring benzimidazole group.

**Figure 3:**
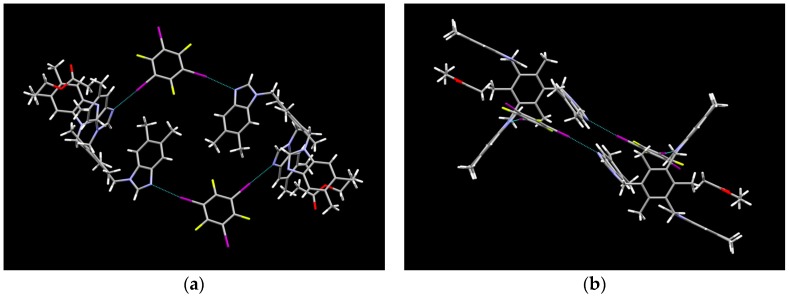
(**a**) Tetramer in the crystal structure of **135XB:E** (**b**) Ethyl acetate wedged between benzimidazole arms.

The structure determination of the product resulting from the reaction between **135XB** and **E** revealed that an ethyl acetate solvate had formed (the overall stoichiometry is 1:1:1), Figure 3. The solvent does not participate in any noteworthy interactions, but rather is lodged within the hydrophobic cavity created by the two benzimidazole arms. The three arms are on the same face of the benzene scaffold. Two of the three halogen atoms on **135XB** participate in C–I⋯N halogen bonds leading to a centrosymmetric tetrameric aggregate. The shorter I⋯N bond takes place with the (N) atom in a perpendicular benzimidazole site, whereas the longer I⋯N contact is formed with the acceptor site pointing away from the central scaffold, rotated along the C–N axis. 

**Figure 4:**
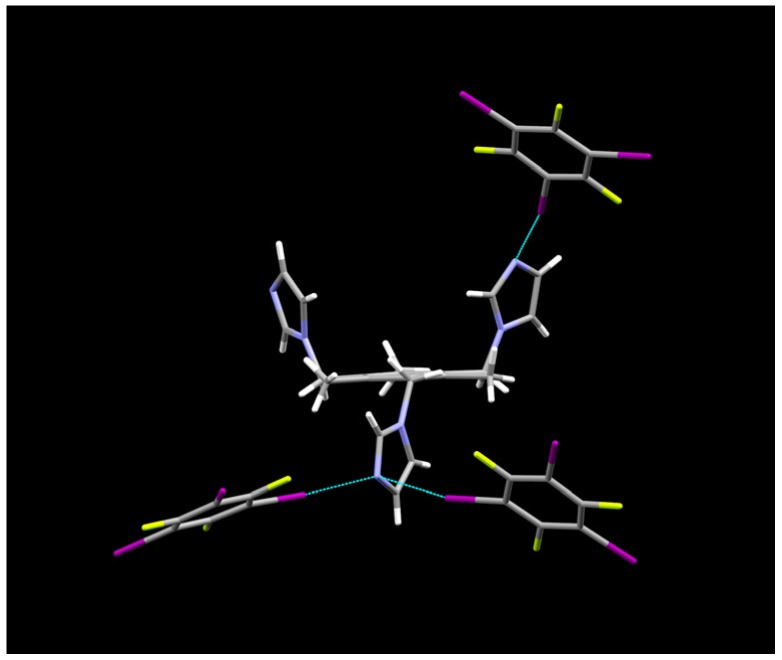
Primary halogen bond interactions in the structure of **135XB:A**.

Finally, in the 1:1 crystal structure of **135XB:A** all three iodine atoms of the donor participate in conventional C–I⋯N halogen bonds, Figure 4. The 1D chains propagate via C–I⋯N bifurcated interactions and is also further extended into 3D molecular networks through a conventional C–I⋯N interaction. The molecular network is further stabilized by bifurcated C–H⋯N interactions. 

### 2.2. Aromatic Stacking

**Figure 5:**
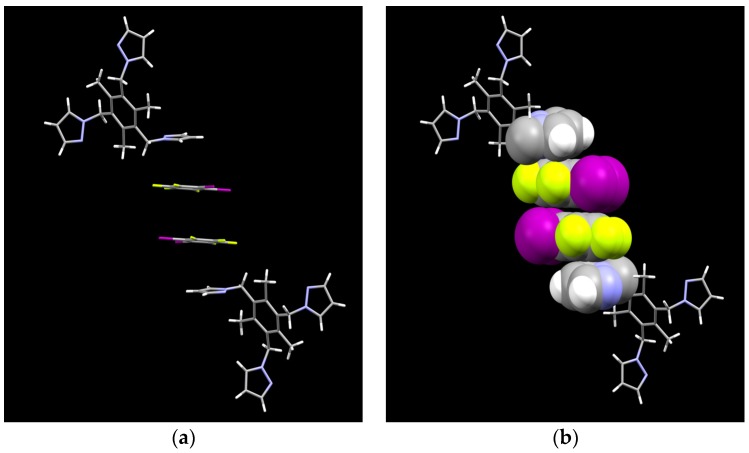
(**a**) Stacking interactions between the two components in the structure of **12XB:B**; (**b**) Space filling representation of the packing showed in (**a**).

In the crystal structure of **12XB:B**, a stacked dimer of donors is sandwiched between two symmetry related arms of acceptor molecules, Figure 5. 

**Figure 6:**
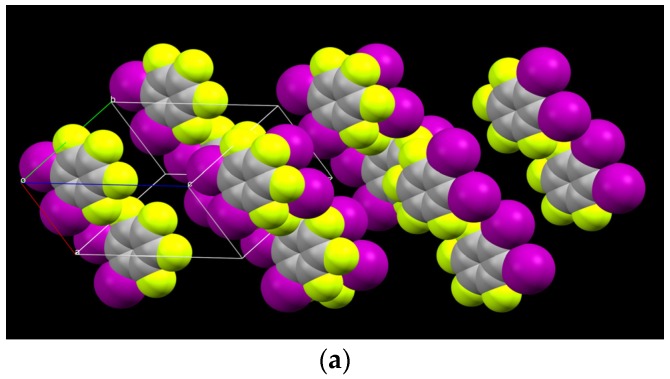

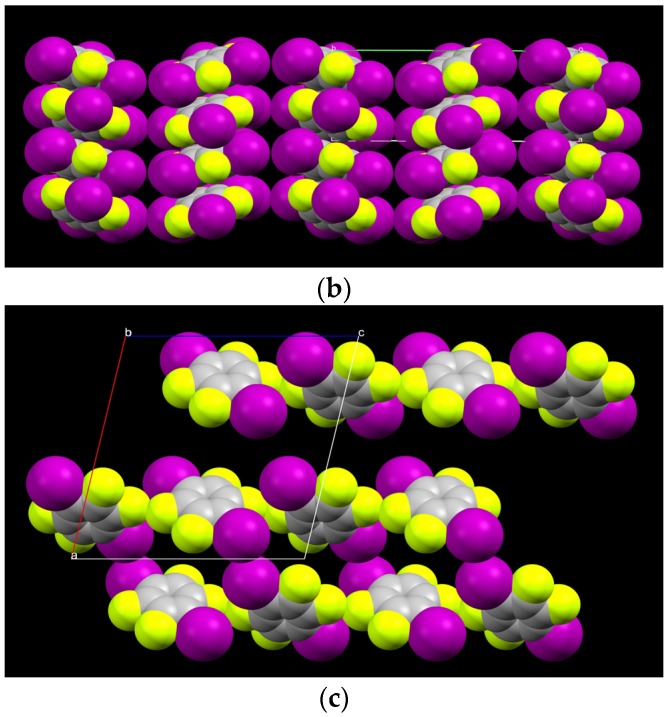
(**a**) Stacking of donors in **12XB:B** (**b**) Stacking of donors in **135XB:A**; (**c**) Close packing of donor molecules in **14XB:E**.

An off-set packing mode or rather a parallel-displaced geometry can be noted between donor molecules **12XB** and **135XB** in co-crystals **12XB:B** and **135XB:A,** respectively, Figure 6. **14XB** in the crystal structure of **14XB:E** display several C⋯F close contacts, which probably arise due to close packing of the donor molecules, Figure 6.

## 3. Discussion

There are no known halogen-bond-based crystal structures for these acceptor molecules although two co-crystals containing a tris-pyridyl acceptor with 1:2 and 1:0.5 stoichiometries with **14XB** and **135XB**, respectively, have been reported [29]. It has been noted on several occasions that it is difficult to control the resulting stoichiometry in co-crystals involving multi-topic halogen-bond donors, such as those employed in this study [26,29]. Therefore, it is not surprising that the intermolecular outcomes in this study did not yield consistent results with regards to satisfying all available XB donor sites. For both co-crystals **12XB:B** and **14XB:E** the targeted stoichiometry was 3:2, however, the observed stoichiometry was 1:1 in both cases. For **135XB:E** and **135XB:A**, a 1:1 stoichiometry was observed as expected, but with rather unexpected donor-acceptor sites. For co-crystal **135XB:E**, only two out of the three halogen atoms form interactions and in co-crystal **135XB:A**, a bifurcated halogen bond and a conventional halogen bond interact with two imidazole sites of the tritopic acceptor. These results clearly underscore the difficulty in controlling the delicate balance between intermolecular interactions. In order to examine this challenge further, a systematic CSD study was carried out in order to extract more structural information on the inability (or reluctance) of multi-topic XB donors to simultaneously engage all donor sites in the solid state. 

We gathered geometric data on C–I⋯N bond lengths and bond angles, as well as the extent of halogen bonding, on all structures of **12XB**, **14XB** and **135XB** involving potential acceptor molecules with accessible *sp*^2^-hybridized nitrogen atoms. First of all, the results indicate that ditopic donors are generally much more likely to engage all their sites in halogen bonding (17/19 for **12XB** and 97/100 for **14XB**, respectively, see Table 1), whereas for the tritopic donor, the success-rate for having a full complement of halogen bonds has dropped to 47% (14/30). Part of the reason may be found in the slightly higher electrostatic potentials which accompanies the ditopic donors (163 kJ/mol for **12XB**, 169 kJ/mol for **14XB**, and 158 for **135XB,** respectively), see Table 1, but the number of available acceptor sites, possible steric considerations, and step-wise deactivation of the donor sites upon binding, are more likely to be the main contributors. 

**Figure 7:**
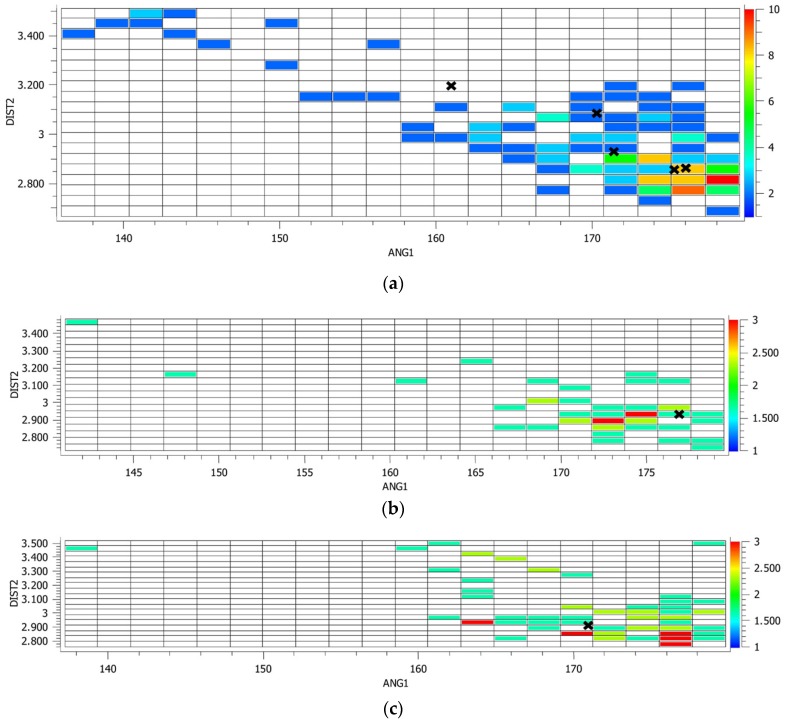
Halogen-bond distances and angles extracted from a CSD search on crystal containing *sp*^2^-hybridized nitrogen atoms as acceptors and (**a**) **135XB**; (**b**) **12XB** and (**c**) **14XB**, respectively. ANG1 = <(C−I⋯N)/°, and DIST2 = d(I⋯N)/Å The colors indicate number of hits in each cell. The “**×**” is correspond to data points from the current study.

There is obviously a difference between the probability that ditopic and tritopic donors will be able to engage in a maximum number of halogen-bonds, which triggered the question as to whether there are any notable differences in halogen-bond metrics between the two types of molecules. To address this issue we constructed three separate graphs where the I⋯N bond length was plotted against the C–I⋯N bond angle for all halogen bonds in crystal structures containing either of the three donors, **12XB, 14XB**, or **135XB**, Figure 7. It is quite notable that the overall appearance of the graphs for the ditopic acceptors is quite different to the way in which the analogous data for **135XB** comes out. First of all, the I⋯N bond-angles for tritopic structures cover a much broader range, and the expected positive correlation between larger (more linear) angles and shorter I⋯N distances is also readily apparent, Figure 7a. For the ditopic donors, the angular dependence has a much more narrow distribution and the vast majority of C–I⋯N bond angles are greater than 160 °C. This underscores that in order for **135XB** to simultaneously form three halogen bonds, the molecule is often forced to make a structural compromise (resulting in considerable deviations from linearity), whereas ditopic halogen-bond donors are more likely to be able to find two suitable acceptors that are both oriented in such a way that two near-linear bonds are produced.

**Figure 8:**
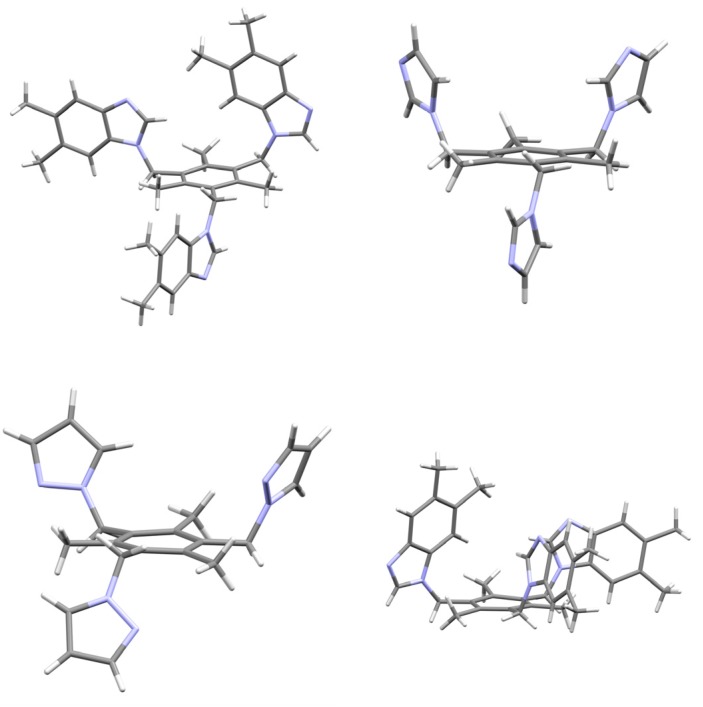
Molecular geometries of the tritopic acceptor molecules in the crystal structures of **14XB:E** (top left), **12XB:B** (bottom left), **135XB:A** (top right), and **135XB:E** (bottom right). The crown conformation is only observed in the crystal structure of **135XB:E**, whereas the chair is present in the other three structures.

Part of the reason for using flexible tritopic acceptor molecules was to maximize the opportunities for formation of geometrically near-linear meaningful C–I⋯N halogen bonds to ditopic and tritopic donors. The tritopic acceptors used in this study contain several rotatable bonds but the molecular geometries can be simplified into one of two classes, a “crown” conformation with all three arms on the same side of the aromatic core, or a “chair” conformation with two arms on one side and the third arm on the opposite side of the aromatic core. As it turns out, both types of conformations were observed in these crystal structures with the chair appearing in the structures of **14XB:E**, **12XB:B**, **135XB:A** and the crown in the crystal structure of **135XB:E**, Figure 8. 

The fact that **E** was found in both types of conformations also means that these molecules are not strongly predisposed to one arrangement over another. Despite this flexibility, it was not possible to realize a perfect match between donors and acceptors in these structures simply by controlling the reaction stoichiometry. A key explanation for this behavior can, undoubtedly, be found by evoking the sequential σ-hole deactivation that takes place in multi-topic halogen bond donors upon binding, as shown by Formigue [27] and van der Boom [26]. In the co-crystals presented herein, it is likely that when C–I⋯N binding interactions takes place, the magnitude of the σ-hole on the non-bonded halogen atom(s) diminish to a point where C–H hydrogen-bond donors suddenly become competitive, resulting in C–H⋯N interactions (as observed in the crystal structure of **14XB:E**, Figure 2).

Even though the halogen bond can deliver selectivity, strength and directionality, much more work is still required before we can fully realize its potential as a reliable synthetic vector capable of delivering supramolecular assemblies with desired chemical composition, stoichiometry, and topology. 

## 4. Materials and Methods

All reagents, solvents, precursors and halogen-bond donors were purchased from commercial sources and were used as received without further purification. A Fisher-Johns melting point apparatus (Vernon Hills, IL, USA) was used to determine melting points. A Varian Unity Plus (400 MHz) NMR spectrophotometer (Varian, Inc. Palo Alto, CA, USA) was used to record nuclear magnetic resonance spectra using the residual solvent signal as a reference. Infrared spectroscopic analyses were performed with a Nicolet 380 FT-IR instrument (Thermo Scientific, Madison, WI, USA).

### 4.1. Molecular Electrostatic Potential Calculations

To calculate the electrostatic potentials of the donor molecules, the geometries were optimized using hybrid density functional B3LYP level of theory with 6–31G* basis set in vacuum. All molecules were geometry optimized with the maxima and minima in the electrostatic potential surface (0.002 e au^−1^ isosurface), determined using a positive point charge in the vacuum as a probe. These numbers, in other words, surface potentials, are the coulombic interaction energies (in kJ·mol^−1^) between the positive point probe and the surface of the molecule at that point. All calculations were done using Spartan 10 software (Wavefunction Inc., Irvine, CA, USA, 2010).

### 4.2. CSD Search

A CSD search on the donor molecules **14XB**, **12XB** and **135XB** were performed using the following constraints: “not disordered”, “no errors”, “not polymeric”, “no ions”, “no powder structures”, “3D coordinates determined” and “only organics”. The search for halogen bonds of the above mention molecules was limited to acceptors with *sp*^2^-hybridized nitrogen atoms. All the CSD search queries were run using ConQuest Version 1.19, CSD 5.38 November 2016 (CCDC, Cambridge, UK).

### 4.3. Synthesis of Tritopic Acceptors

#### 4.3.1. Synthesis of 1,3,5-Tris(bromomethyl)-2,4,6-trimethylbenzene (**α**) 

To a mixture of mesitylene (12.0 g; 0.10 mol), paraformaldehyde (10.26 g; 0.34 mol), and 75 mL of glacial acetic acid, 75 mL of a 33 *wt*% HBr/acetic acid solution was added rapidly. The mixture was kept for 12 h at 95 °C and then poured into 100 mL of water. The product was filtered off on a Buchner funnel and dried. Flash column chromatography (hexane: ethyl acetate = 98.5:1.5) gave the desired product as colorless needles. 90% yield. mp 186 °C (lit. [30] mp 183–186 °C); ^1^H-NMR (δH; CDCl_3_, 400 MHz) δ 4.58 (s, 6H), 2.46 (s, 9H).

#### 4.3.2. Synthesis of 1,3,5-Tris(imidazole-1-yl-methyl)-2,4,6-trimethylbenzene (**A**)

To a mixture of imidazole (874 mg, 12.8 mmol) in acetonitrile (50 mL), in a 100 mL round-bottomed flask, NaOH (1.029 g, 25.72 mmol) was added and stirred at room temperature for two hours. 1,3,5-Tris(bromomethyl)-2,4,6-trimethylbenzene (1.416 g, 4.000 mmol) in acetonitrile (20 mL) was added to the reaction mixture and refluxed for 24 h at 50–60 °C. The reaction mixture was monitored with TLC and after completion, the solvent was removed by rotary evaporation, the residue was dissolved in water (100 mL) and extracted with methylene chloride (30 mL × 5). The organic layers were combined, dried over anhydrous MgSO_4_, filtered and purified by flash column chromatography (CH_2_Cl_2_/MeOH = 5/1) to give the desired product as a white solid. Recrystallization in ethyl acetate produced clear block like crystals 64% yield. mp 214–215 °C (Lit value 226–227 °C) [31]; ^1^H-NMR (δH; CDCl_3_, 400 MHz) δ 7.31 (s, 3H), 7.07 (s, 3H), 6.75 (s, 3H), 5.24 (s, 6H) 2.32 (s, 9H).

#### 4.3.3. Synthesis of 1,3,5-Tris(pyrazole-1-yl-methyl)-2,4,6-trimethylbenzene (**B**) 

To a mixture of pyrazole (874 mg, 12.8 mmol) in acetonitrile (50 mL), in a 100 mL round-bottomed flask, 60% NaH (1.029 g, 25.72 mmol) was added and stirred at room temperature for two hours. 1,3,5-Tris(bromomethyl)-2,4,6-trimethylbenzene (1.416 g, 4.000 mmol) in acetonitrile (20 mL) was added to the reaction mixture and refluxed for 24 h. The reaction mixture was monitored with TLC and after completion, the solvent was removed by rotary evaporation, the residue was dissolved in water (100 mL) and extracted with methylene chloride (30 mL × 5). The organic layers were combined, dried over anhydrous MgSO_4_, filtered and purified by flash column chromatography (CH_2_Cl_2_/MeOH = 10/1) to give the desired product as a white solid. 53% yield. mp 132–133 °C (Lit value 130–131 °C) [32]; ^1^H-NMR (δH; CDCl_3_, 400 MHz) δ 7.53 (d, 3H), 7.06 (d, 3H), 6.21 (t, 3H), 5.44 (s, 6H), 2.35 (s, 9H).

#### 4.3.4. Synthesis of 1,3,5-Tris(3,5-dimethylpyrazole-1-yl-methyl)-2,4,6-trimethylbenzene (**C**)

To a mixture of 3,5-dimethylpyrazole (1.230 g, 12.8 mmol) in acetonitrile (50 mL), in a 100 mL round-bottomed flask, 60% NaH (1.029 g, 25.72 mmol) was added and stirred at room temperature for two hours. 1,3,5-Tris(bromomethyl)-2,4,6-trimethylbenzene (1.416 g, 4.000 mmol) in acetonitrile (20 mL) was added to the reaction mixture and refluxed for 24 h. The reaction mixture was monitored with TLC and after completion, the solvent was removed by rotary evaporation, the residue was dissolved in water (100 mL) and extracted with methylene chloride (30 mL × 5). The organic layers were combined, dried over anhydrous MgSO_4_, filtered and purified by flash column chromatography (CH_2_Cl_2_/MeOH = 10/1) to give the desired product as a white solid. 61% yield. mp 245–247 °C (Lit value 248–250 °C) [32]; ^1^H-NMR (δH; CDCl_3_, 400 MHz) δ 5.75 (s, 3H), 5.18 (s, 6H), 2.23 (s, 9H), 2.13 (s, 9H), 2.11 (s, 9H).

#### 4.3.5. Synthesis of 1,3,5-Tris(benzimidazole-1-yl-methyl)-2,4,6-trimethylbenzene (**D**)

To a mixture of benzimidazole (1.512 g, 12.8 mmol) in THF (50 mL), in a 100 mL round-bottomed flask, KOH (1.029 g, 25.72 mmol) was slowly added and stirred at room temperature under N_2_ gas. After about 4 h, a solution of 1,3,5-Tris(bromomethyl)-2,4,6-trimethyl benzene (1.416 g, 4.000 mmol) in 50 mL of THF was added dropwise, and the reaction mixture was stirred continuously overnight. The solvent was subsequently removed under reduced pressure, and the residue was poured into 100 mL of water and extracted 3 times with dichloromethane (3 × 50 mL). The combined organic extracts were washed with water, dried (MgSO_4_), and concentrated. The crude product was purified by recrystallization from hot ethanol to produce colorless plates. 78% Yield. mp > 300 °C (Lit value > 300 °C) [31]; ^1^H-NMR (δH; CDCl_3_, 400 MHz) δ 7.84 (m, 3H), 7.46 (s, 3H), 7.41 (m, 3H), 7.34 (m, 6H), 5.42 (s, 3H) 2.33 (s, 3H).

#### 4.3.6. Synthesis of 1,3,5-Tris(5,6-dimethylbenziimidazole-1-yl-methyl)-2,4,6-trimethylbenzene (**E**)

To a mixture of 5,6-dimethylbenzimidazole (1.871 g, 12.8 mmol) in THF (50 mL), in a 100 mL round-bottomed flask, KOH (1.029 g, 25.72 mmol) was slowly added and stirred at room temperature under N_2_ gas. After about 4 h, a solution of 1,3,5-Tris(bromomethyl)-2,4,6-trimethyl benzene (1.416 g, 4.000 mmol) in 50 mL of THF was added dropwise, and the reaction mixture was stirred continuously overnight. The solvent was subsequently removed under reduced pressure, and the residue was poured into 100 mL of water and extracted 3 times with dichloromethane (3 × 50 mL). The combined organic extracts were washed with water, dried (MgSO_4_), and concentrated. The crude product was purified by recrystallization from hot ethanol which yielded pale yellowish-green pyramid-like crystals. 81% Yield. mp 285–290 °C (Lit value > 280 °C) [33]; ^1^H-NMR (δH; CDCl_3_, 400 MHz) δ 7.58 (s, 3H), 7.33 (s, 3H), 7.20 (s, 3H) 5.33 (s, 6H), 2.42 (s, 9H), 2.39 (s, 9H), 2.30 (s, 9H).

#### 4.3.7. Synthesis of 1,3,5-Tris(bromomethyl)benzene (**β**)

A mixture of mesitylene (2.8 mL, 20 mmol), *N*-bromosuccinimide (10.62 g, 60 mmol), and benzoyl peroxide (0.11 g) in CCl_4_ (30 mL) was stirred and heated under N_2_ for 14 h at 90 °C. The reaction mixture was monitored with TLC and after completion, the solution was cooled in an ice bath and the succinimide was filtered off and washed with carbon tetrachloride. The filtrate was washed with water and dried over anhydrous MgSO_4_. Upon concentration of the CCl_4_ solution, a pale-yellow solid was obtained. Recrystallization in a 1:1 mixture of ethanol/hexane afforded colourless needle-like crystals. 81% Yield. mp 87–89 °C (Lit value 86–87 °C) [34]; ^1^H-NMR (δH; CDCl_3_, 400 MHz) δ 7.36 (s, 3H), 4.46 (s, 6H).

The synthesis of **A′**, **B′**, **C′**, **D′** and **E’** were carried out using the same experimental conditions as employed for their analogues.

#### 4.3.8. Synthesis of 1,3,5-Tris(imidazole-1-yl-methyl)benzene (**A′**)

The desired product was obtained as a white solid. 54% yield. mp 173–175 °C (Lit value 175–179 °C) [35]; ^1^H-NMR (δ_H_; CDCl_3_, 400 MHz) δ 7.52 (s, 3H), 7.12 (s, 3H), 6.85 (m, 6H) 5.07 (s, 6H).

#### 4.3.9. Synthesis of 1,3,5-Tris(pyrazole -1-yl-methyl)benzene (**B′**)

The desired product was obtained as an off-white solid. 55% yield. mp 63–65 °C (Lit value 74–76 °C, 60–61 °C) [32]; ^1^H-NMR (δ_H_; CDCl_3_, 400 MHz) δ 7.52 (d, 3H), 7.34 (d, 3H), 6.91 (s, 6H) 6.27 (t, 6H), 5.24 (s, 6H).

#### 4.3.10. Synthesis of 1,3,5-Tris(3,5-dimethylpyrazole -1-yl-methyl)benzene (**C′**)

The desired product was obtained as an off-white solid. 58% yield. mp 94–96 °C ^1^H-NMR (δ_H_; CDCl_3_, 400 MHz) δ 6.56 (s, 3H), 5.81 (s, 3H), 5.09 (s, 6H) 2.22 (s, 9H), 2.06 (s, 9H).

#### 4.3.11. Synthesis of 1,3,5-Tris(benzimidazole -1-yl-methyl)benzene (**D′**)

The desired product was obtained upon recrystallization in ethyl acetate to produce colorless rod-like crystals. 71% yield. mp 102–104 °C (Lit value 229–231 °C) [36]; ^1^H-NMR (δ_H_; CDCl_3_, 400 MHz) δ 7.92 (s, 3H), 7.84 (d, 3H), 7.31 (t, 3H), 7.21 (t, 3H), 7.06 (d, 3H), 7.06 (d, 3H), 6.91 (s, 3H), 5.25 (s, 6H),

#### 4.3.12. Synthesis of 1,3,5-Tris(5,6-dimethylbenziimidazole -1-yl-methyl)benzene (**E′**)

The desired product was obtained as a white solid. 76% yield. mp 295–296 °C ^1^H-NMR (δ_H_; CDCl_3_, 400 MHz) δ 7.78 (s, 3H), 7.58 (s, 3H), 6.87 (s, 3H), 6.85 (s, 3H) 5.18 (s, 6H), 2.37 (s, 9H), 2.28 (s, 9H).

### 4.4. Grinding Experiments

Screenings for co-crystals were performed using solvent-assisted grinding technique. In a typical grinding experiment, the stoichiometric amounts (refer to supplementary information) of donor and acceptor were mixed in a microwell with the aid of a pestle and a drop of solvent (methylene chloride). The resulting solids from each reaction were subjected to IR analysis for characterization. A successful interaction would be characterized by specific peak shifts observed in the ground mixture compared to the starting compounds.

### 4.5. Synthesis of Co-Crystals

All co-crystal growth experiments were carried out from the resulting solid mixtures used in the grinding experiments via slow evaporation of a 1:1 mixture of methylene chloride: ethyl acetate. A model experiment would entail, transferring the ground mixture into a 2-dram glass vial, which was then fully dissolved in a minimal amount of solvent mixture. The loosely capped vial was left undisturbed at ambient conditions to allow the solvent to evaporate slowly. Out of 40 experiments, four produced crystals suitable for single-crystal X-ray diffraction.

### 4.6. X-ray Crystallography

Upon preliminary IR and melting point analysis, crystals were subjected to single crystal X-ray diffraction (CCDC 1587213-1587216). These data can be obtained free of charge via http://www.ccdc.cam.ac.uk/conts/retrieving.html (or from the CCDC, 12 Union Road, Cambridge CB2 1EZ, UK; Fax: +44-1223-336033; E-mail: deposit@ccdc.cam.ac.uk). Experimental details are recorded in the ESI. 

## 5. Conclusions

To learn more about the use of halogen bonds as a practical tool for predictable crystal engineering and supramolecular synthetic chemistry, a series of ten tritopic *N*-heterocyclic compounds were combined with four different multi-topic halogen-bond donors in attempted co-crystallizations. Initial screening using IR spectroscopy for analyzing the outcome of each reaction showed that over 70% of the 40 experiments produced halogen-bonded co-crystals. The crystal structures of four of them were obtained. 

The XB bonded co-crystal structures show two different architectures: discrete tetrameric aggregates and 1D chains. A comparison with data from a systematic CSD analysis shows that the halogen-bond distances and angles in the four structures presented herein are consistent with commonly observed parameters. In addition, a variety of π-stacking and C–H⋯π interactions were also seen in two co-crystals. Our results underscore the difficulty of controlling stoichiometries and chemical compositions of targeted products when attempting to synthesize co-crystals with reactants that carry multiple donor- and acceptor sites. Only two of the four co-crystals met the expected stoichiometric ratios, even though they also displayed unexpected connectivities. A key factor that contributes to the synthetic challenges is the fact that upon deactivation of σ-hole and halogen-bond donor capability, other interactions, such as C–H hydrogen-bond donors become competitive, which subsequently leads to a preference for C–H⋯N hydrogen bonds over C–I⋯N halogen bonds. 

## Figures and Tables

**Table 1:** CSD-based summary of frequency of interactions to available halogen-bond donors and MEPs values.

Halogen Bond Donor	135XB		12XB		14XB	
MEP	158 kJ/mol		163 kJ/mol		169 kJ/moL	
No. Hits in the CSD	30 hits		19 hits		100 hits	
Coordination	2 out of 3	3 out of 3	1 out of 2	2 out of 2	1 out of 2	2 out of 2
Result	16	14	2	17	3	97
% outcome	53%	47%	10.5%	89.5%	3%	97%

**Table 2:** Grinding results.

		Acceptors										%Success
		A	A′	B	B′	C	C′	D	D′	E	E′	
Donors	**135XB**	√	√	√	√	√	√	√	√	√	√	100
	**12XB**	-	√	√	√	√	√	-	√	√	√	80
	**14XB**	√	√	√	√	-	-	-	-	√	√	60
	**44XB**	-	-	√	-	√	-	√	√	√	-	50
%Success		50	75	100	75	75	50	50	75	100	75	

**Table 3:** Crystallographic data.

Code	14XB:E	135XB:E	12XB:B	135XB:A
Formula moiety	(C_39_H_42_N_6_), (C_6_F_4_I_2_)	(C_39_H_42_N_6_), (C_6_F_3_I_3_), (C_4_H_8_O_2_)	(C_21_H_24_N_6_), (C_6_F_4_I_2_)	(C_21_H_24_N_6_), (C_6_F_3_I_3_)
Empirical formula	C_45_H_42_N_6_F_4_I_2_	C_49_H_50_F_3_I_3_N_6_O_2_	C_27_H_24_N_6_F_4_I_2_	C_27_H_24_F_3_I_3_N_6_
Molecular weight	996.65	1192.65	762.32	870.22
Crystal system	Monoclinic	Triclinic	Triclinic	Orthorhombic
Space group	*P*2_1_/*c*	P ī	P ī	*Pbca*
*a*, Å	16.337(6)	9.764(4)	9.255(3)	7.931(2)
*b*, Å	16.340(5)	11.594(4)	11.995(4)	20.310(5)
*c*, Å	15.642(5)	22.901(9)	13.507(5)	37.342(10)
*α*, °	90	101.90(2)	78.82(2)	90
*β*, °	102.862(13)	97.56(3)	84.15(2)	90
*γ*, °	90	99.98(2)	68.959(19)	90
Volume, Å^3^	4071(2)	2460.4(16)	1372.1(8)	296(2)
*Z*	4	2	2	8
Density, g/cm^3^	1.626	1.610	1.845	1.922
*T,*K	130	180	200	296(2)
X-ray wavelength, Å	0.71073	0.71073	0.71073	0.71073
*µ,* mm^−1^	1.604	1.961	2.348	3.164
R_1_ (observed)	0.0442	0.0338	0.0497	0.0638
wR_2_ (all)	0.1198	0.1100	0.1773	0.1740

**Table 4:** Halogen-bond geometries.

Compound	D–I⋯A	d(I⋯A)	d(D⋯A)	<(D–I⋯A)	Symmetry Operation
**12XB:B**	C(29)–I(35)u⋯N(9)v	2.934(7)	5.03(1)	177.0(2)	u = *x,y,z;* v = *x,y,z*
Co-crystal					
**14XB:E**	C(46)–I(52)u⋯N(10)v	2.922(4)	5.024(6)	171.3(2)	u = *x,y,z;* v = *x,y,z*
Co-crystal					
**135XB:E**	C(48)–I(54)u⋯N(23)v	2.936(5)	5.063(7)	171.8(1)	u = *x,y,z;* v = *−x,1 − y,2 − z*
Co-crystal	C(50)–I(56)u⋯N(10)v	2.864(4)	4.991(6)	175.7(1)	u = *x,y,z;* v = *−1 + x,1 + y,z*
Solvate					
**135XB:A**	C(32)–I(38)u⋯N(17)v	2.869(9)	4.95(1)	175.9(3)	u = *x,y,z;* v = *1 + x,y,z*
Co-crystal	C(28)–I(34)u⋯N(17)v	3.206(9)	5.23(1)	162.7(3)	u = *x,y,z;* v = *−1/2 + x, 3/2 − y,1 − z*
	C(30)–I(36)u⋯N(24)v	3.093(2)	5.15(2)	170.9(4)	u = *x,y,z;* v = *½ − x,−1/2 + y,z*

## Supplementary Materials

NMR spectra, IR data, crystallographic information, and thermal data are available online.
