# Supplementary material for: Structural Examination of Halogen-Bonded Co-Crystals of Tritopic Acceptors
Source: Molecules. 2018 Jan 13;23(1):163. doi: 10.3390/molecules23010163 (PMC6017714; doi:10.3390/molecules23010163)
Supplement: Supplementary file 1 [file molecules-23-00163-s001.pdf]

Supporting Information

Structural Examination of Halogen-Bonded Co-Crystals of Tritopic Acceptors

Stefan N. L. Andree[1], Abhijeet Sinha[1] and Christer B. Aakeröy[1*]
1 Department of Chemistry, Kansas State University, Manhattan, KS, 66506; snlandree@ksu.edu
* Correspondence: aakeroy@ksu.edu; Tel.: +1-785-532-6096

Contents

1. NMR Spectra 3
2. IR Data 9
3. Crystallographic Data 11
4. Melting Points 14

1. NMR Spectra

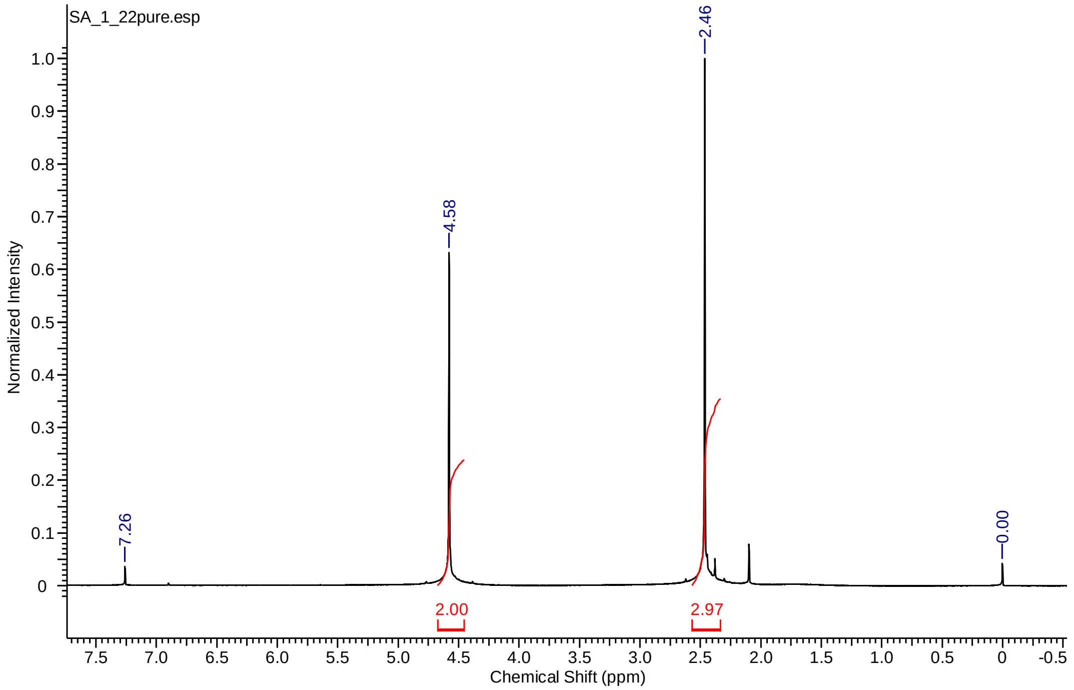

Figure S1: NMR spectrum of 1,3,5-tris(bromomethyl)-2,4,6-trimethyl benzene (α)

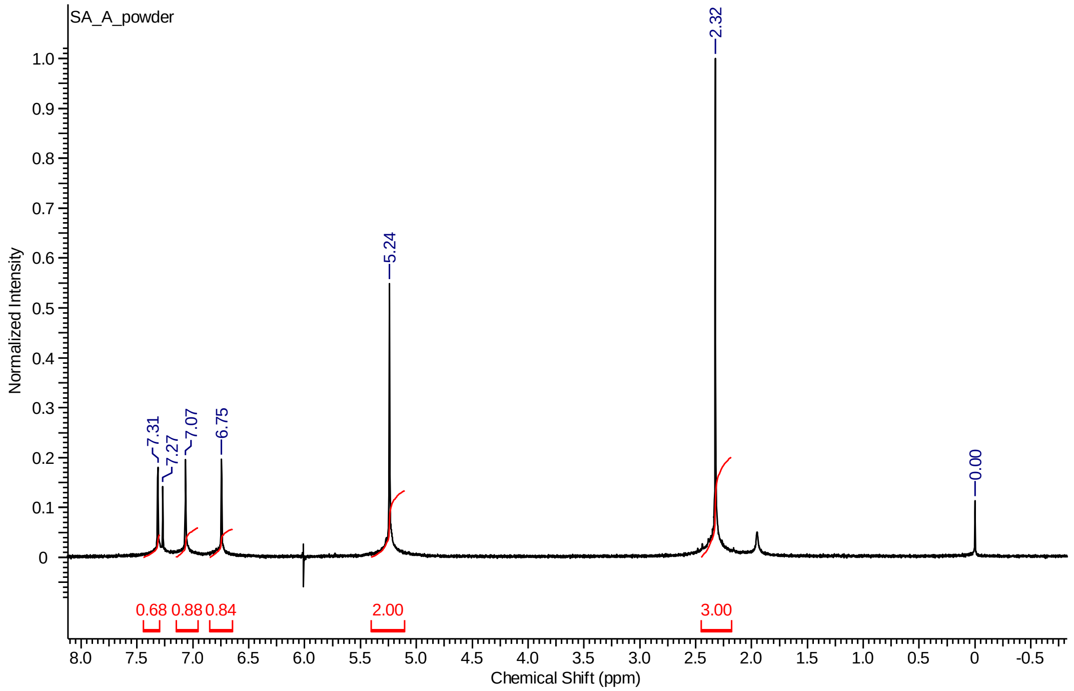

Figure S2: NMR spectrum of 1,3,5-tris(imidazole-1-yl-methyl)-2,4,6-trimethyl benzene (A)

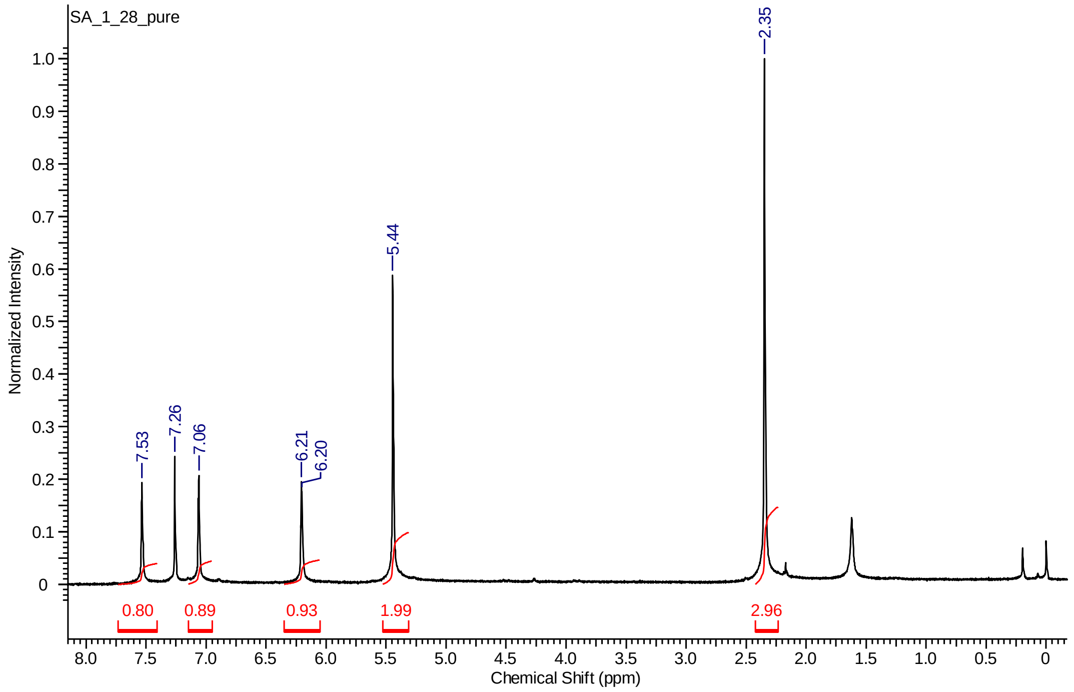

Figure S3: NMR spectrum of 1,3,5-tris(pyrazole-1-yl-methyl)-2,4,6-trimethyl benzene (B)

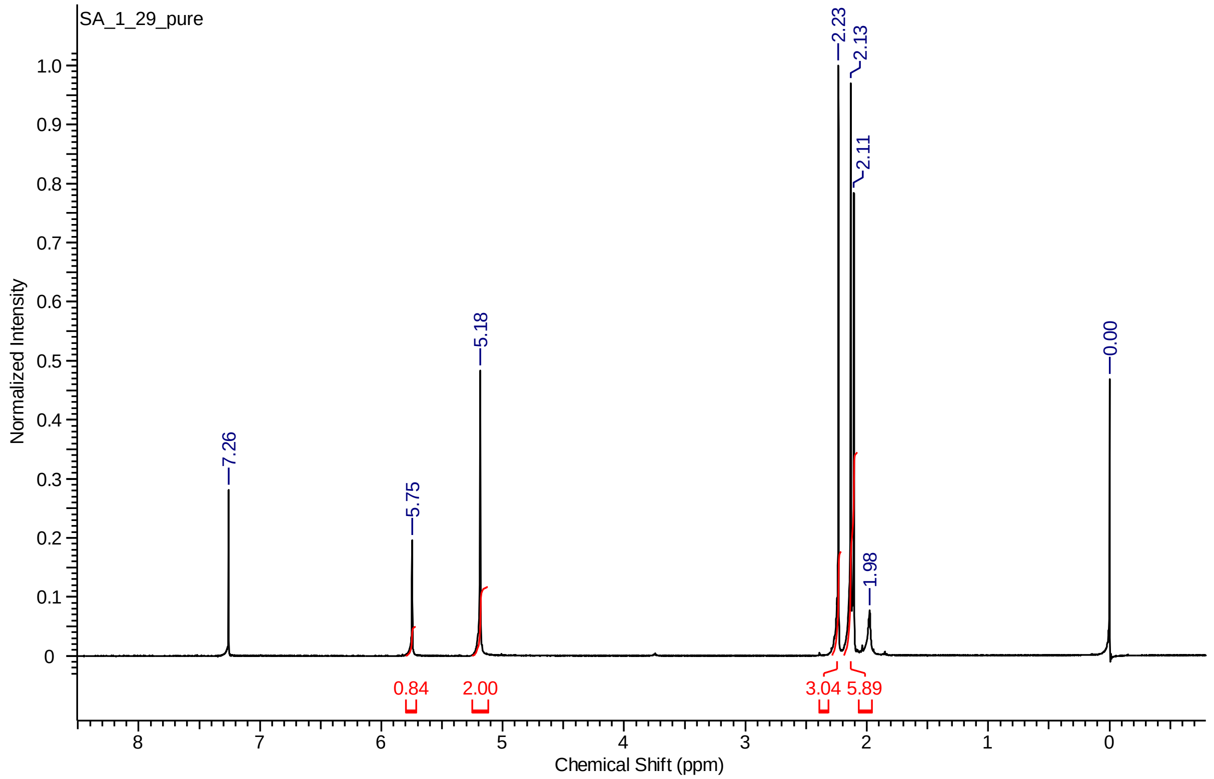

Figure S4: NMR spectrum of 1,3,5-tris(3,5-dimethylpyrazole-1-yl-methyl)-2,4,6-trimethyl benzene (C)

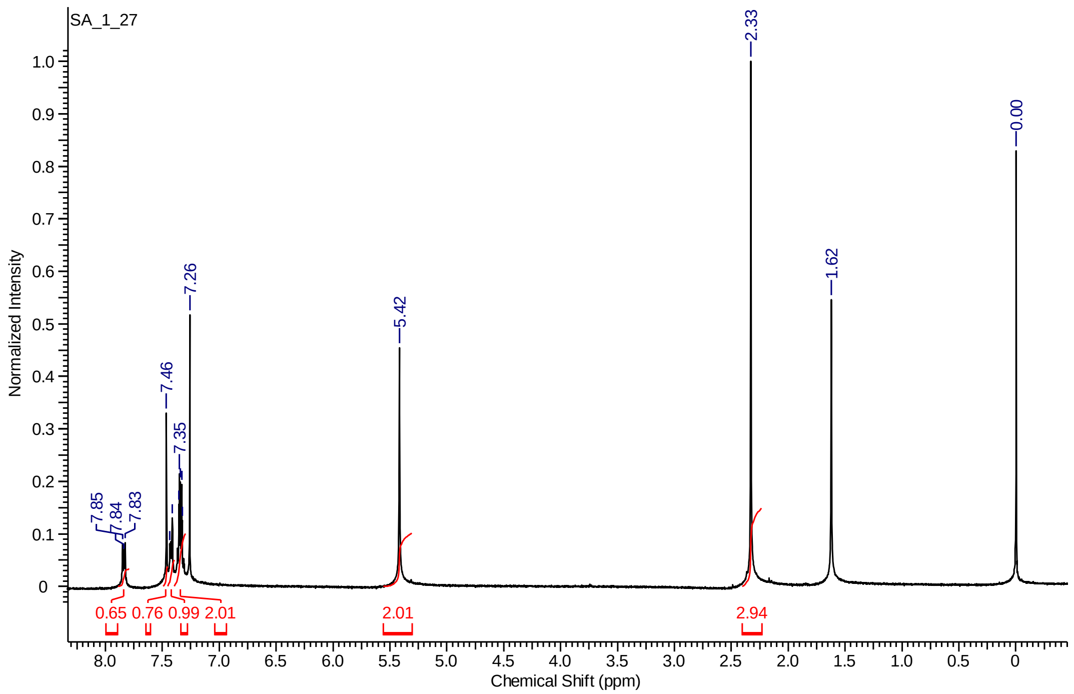

Figure S5: NMR spectrum of 1,3,5-tris(benzimidazole-1-yl-methyl)-2,4,6-trimethyl benzene (D)

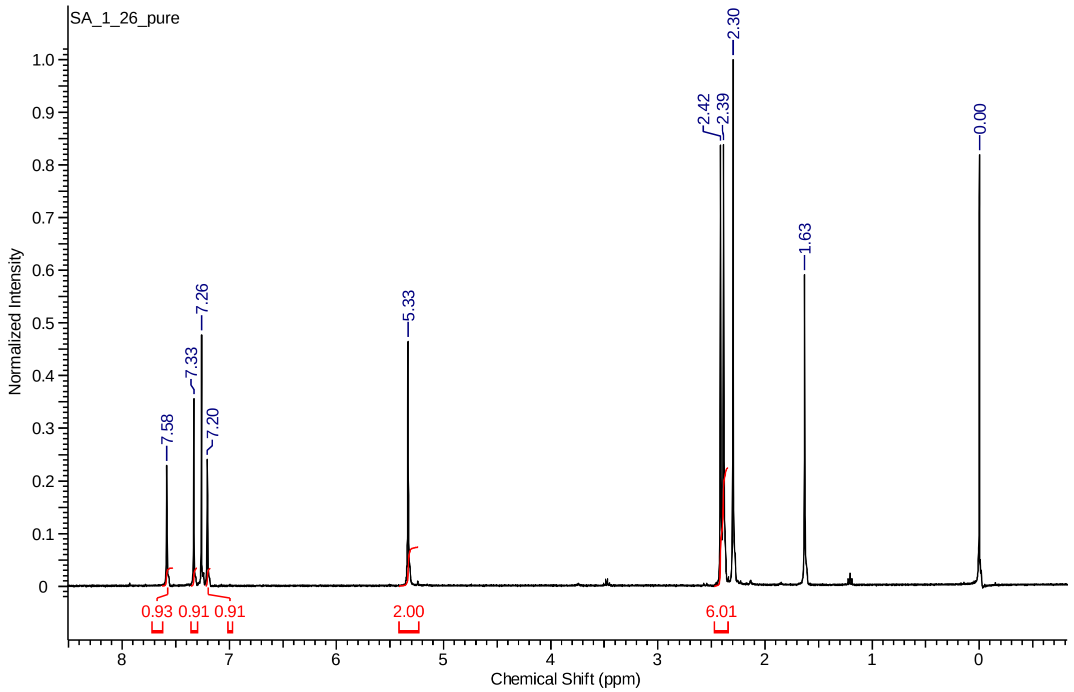

Figure S6: NMR spectrum of 1,3,5-tris(5,6-dimethylbenziimidazole-1-yl-methyl)-2,4,6-trimethyl benzene (E)

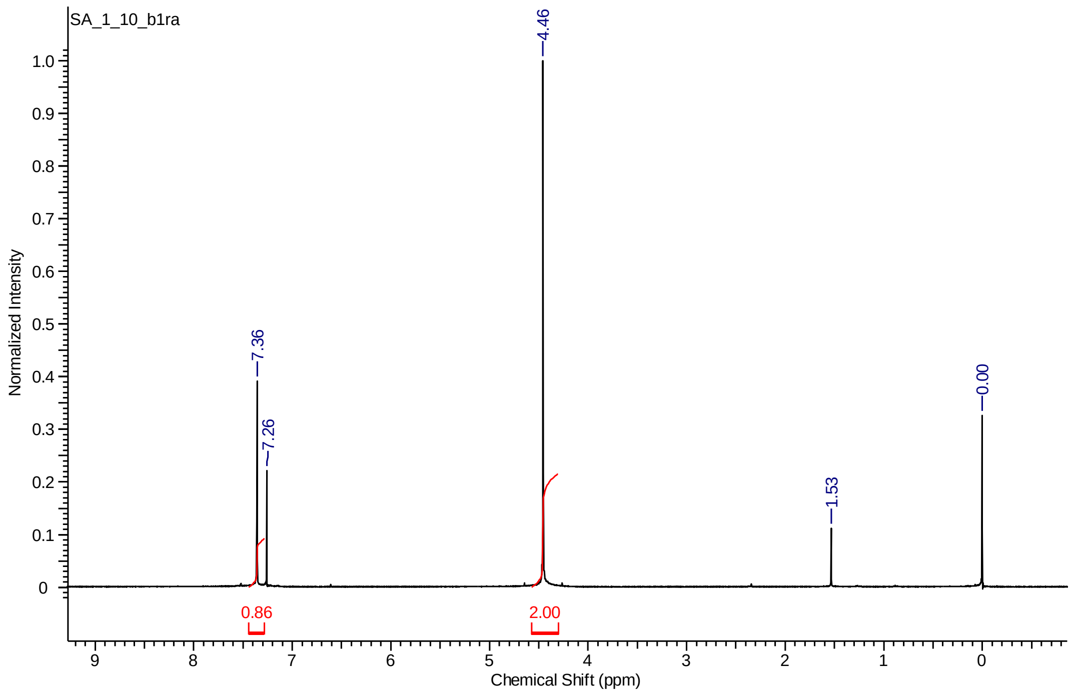

Figure S7: NMR spectrum of 1,3,5-tris(bromomethyl) benzene (β)

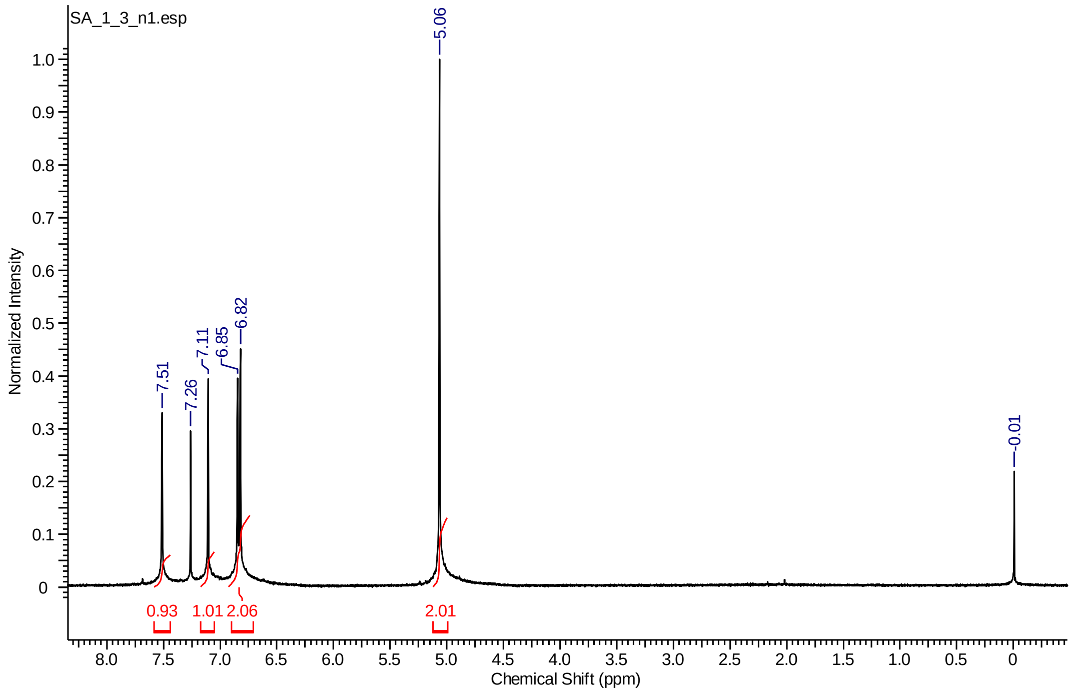

Figure S8: NMR spectrum of 1,3,5-tris(imidazole-1-yl-methyl) benzene (A′)

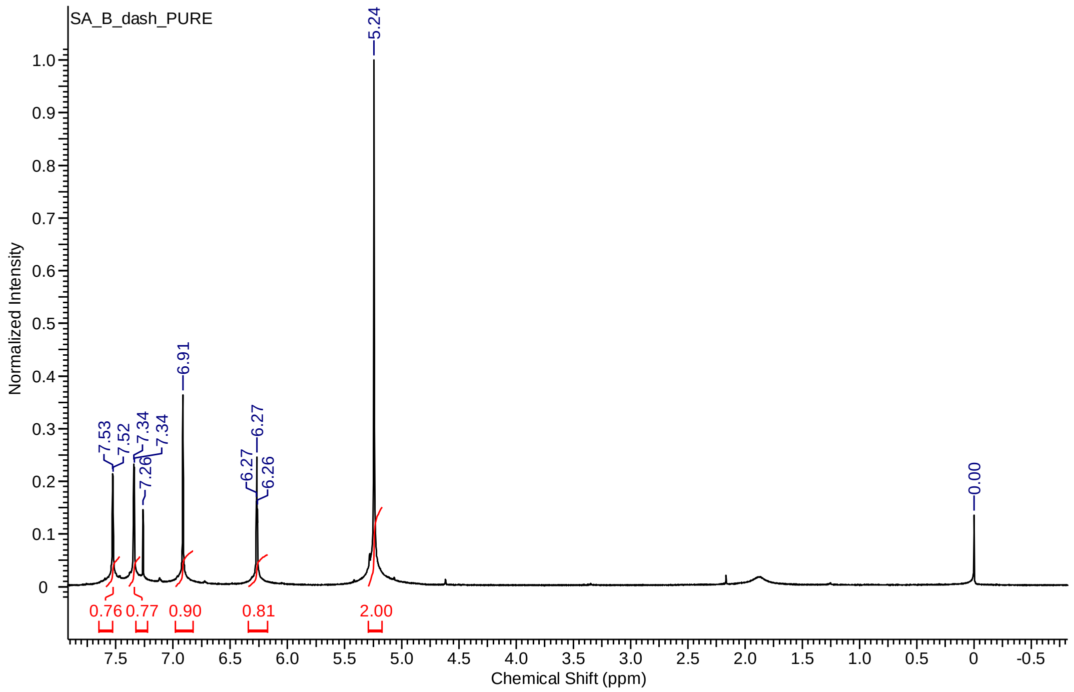

Figure S9: NMR spectrum of 1,3,5-tris(pyrazole -1-yl-methyl) benzene (B′)

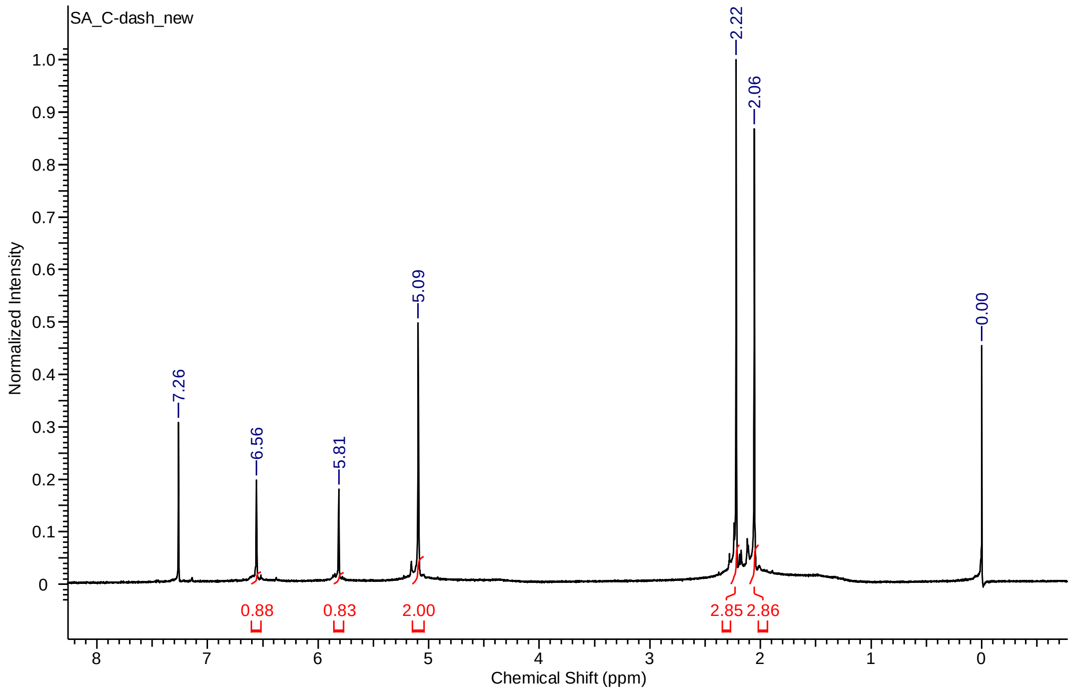

Figure S10: NMR spectrum of 1,3,5-tris(3,5-dimethylpyrazole -1-yl-methyl) benzene (C′)

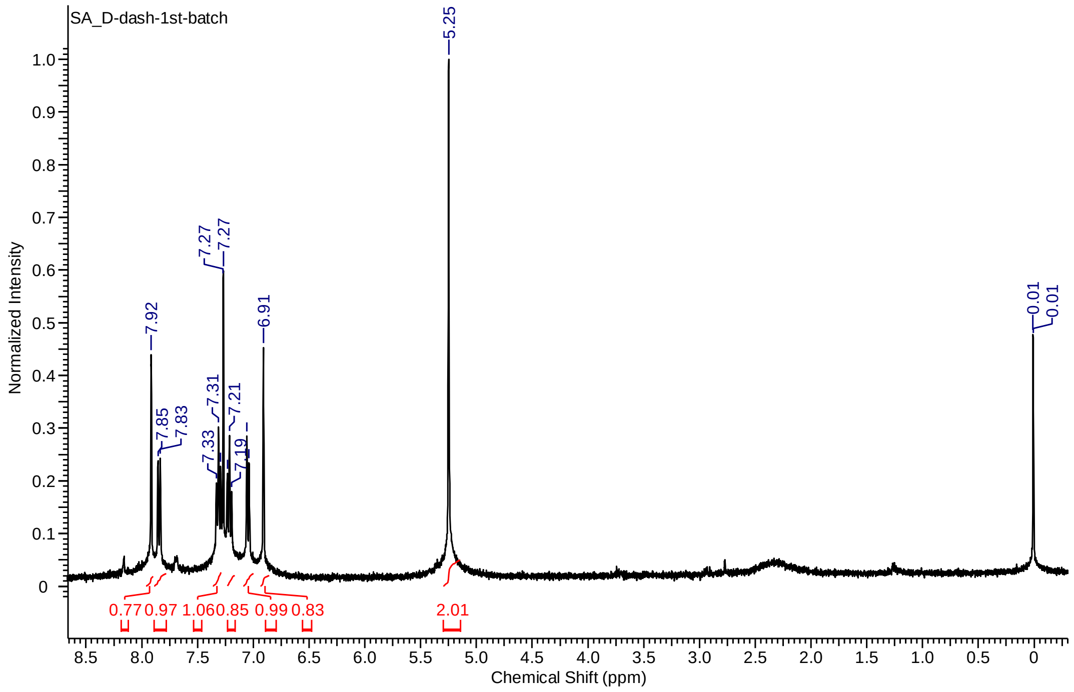

Figure S11: NMR spectrum of 1,3,5-tris(benzimidazole -1-yl-methyl) benzene (D′)

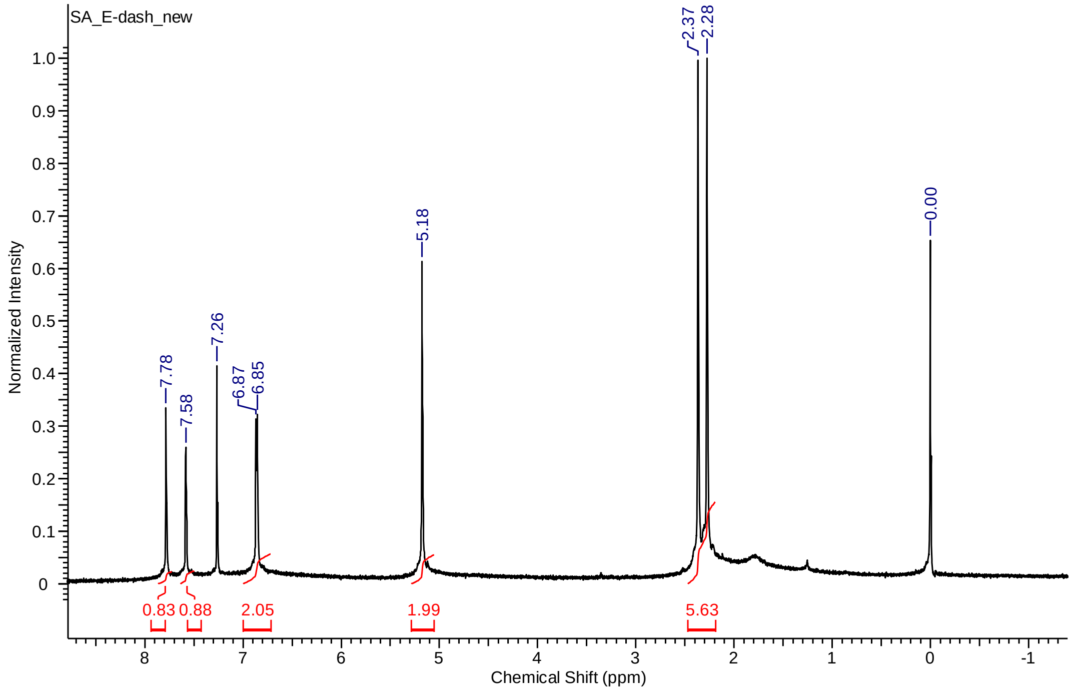

Figure S12: NMR spectrum of 1,3,5-tris(5,6-dimethylbenziimidazole -1-yl-methyl) benzene (E′)

2. IR Data

Table S1: Summary – IR results

Ground mixture ID	Stoichiometry	IR results (cm^{-1})	
		Halogen bond donor	Grinding mixture
14XB:A	3:2	1456, 938	1453, 936
14XB:B	3:2	1456, 938	1460, 940
14XB:C	3:2	1456, 938	1457, 940
14XB:D	3:2	1456, 938	1456, 940
14XB:E	3:2	1456, 938	1461, 941
14XB:A′	3:2	1456, 938	1452, 938
14XB:B′	3:2	1456, 938	1459, 942
14XB:C′	3:2	1456, 938	1456, 939
14XB:D′	3:2	1456, 938	1456, 939
14XB:E′	3:2	1456, 938	1459, 941
12XB:A	3:2	1487, 1436	1486, 1436
12XB:B	3:2	1487, 1436	1485, 1438
12XB:C	3:2	1487, 1436	1484, 1436
12XB:D	3:2	1487, 1436	1485, 1436
12XB:E	3:2	1487, 1436	1482, 1433
12XB:A′	3:2	1487, 1436	1485, 1433
12XB:B′	3:2	1487, 1436	1484, 1435
12XB:C′	3:2	1487, 1436	1484, 1436
12XB:D′	3:2	1487, 1436	1485, 1434
12XB:E′	3:2	1487, 1436	1484, 1433
135XB:A	1:1	1563, 1403, 1049	1561, 1397, 1041
135XB:B	1:1	1563, 1403, 1049	1562, 1392, 1037
135XB:C	1:1	1563, 1403, 1049	1551, 1397, 1042
135XB:D	1:1	1563, 1403, 1049	1560, 1403, 1050
135XB:E	1:1	1563, 1403, 1049	1566, 1398, 1049
135XB:A′	1:1	1563, 1403, 1049	1562, 1399, 1048
135XB:B′	1:1	1563, 1403, 1049	1562, 1392, 1037
135XB:C′	1:1	1563, 1403, 1049	1551, 1397, 1042
135XB:D′	1:1	1563, 1403, 1049	1559, 1400, 1039
135XB:E′	1:1	1563, 1403, 1049	1562, 1398, 1037
44XB:A	3:2	1460, 950	1458, 949
44XB:B	3:2	1460, 950	1466, 957
44XB:C	3:2	1460, 950	1467, 956
44XB:D	3:2	1460, 950	1469, 956
44XB:E	3:2	1460, 950	1469, 957

44XB:A′	3:2	1460, 950	1459, 952
44XB:B′	3:2	1460, 950	1459, 950
44XB:C′	3:2	1460, 950	1458, 951
44XB:D′	3:2	1460, 950	1458, 953
44XB:E′	3:2	1460, 950	1459, 949

3. Crystallographic Data

Crystallography Experimental Details

Datasets were collected on a Bruker Kappa APEX II system using MoKα radiation. Data were collected using APEX2 software.[i] Initial cell constants were found by small widely separated "matrix" runs. Data collection strategies were determined using COSMO.[ii] Scan speed and scan widths were chosen based on scattering power and peak rocking curves. Datasets were collected at 23 ℃ (**SA1707**), -73 ℃ (**SA1704**), -93 ℃ (**SA1603**), and -143 ℃ (**SA1602**) using an Oxford Cryostream low-temperature device.

The unit cell constants and orientation matrix were improved by least-squares refinement of reflections thresholded from the entire dataset. Integration was performed with SAINT,[iii] using this improved unit cell as a starting point. Precise unit cell constants were calculated in SAINT from the final merged dataset. Lorenz and polarization corrections were applied. Multi-scan absorption corrections were performed with SADABS.[iv]

The data were reduced with SHELXTL.[v] The structures were solved in all cases by direct methods without incident. All hydrogen atoms were located in idealized positions and were treated with a riding model. All non-hydrogen atoms were assigned anisotropic thermal parameters. Refinements continued to convergence, using the recommended weighting schemes.

[i] APEX2 v2013.10-0, © 2013, Bruker Analytical X-ray Systems, Madison, WI.

[ii] COSMO v1.61, © 1999 - 2009, Bruker Analytical X-ray Systems, Madison, WI.

[iii] SAINT v8.34a, © 1997 - 2013, Bruker Analytical X-ray Systems, Madison, WI.

[iv] SADABS v2012/1, © 2012, Bruker Analytical X-ray Systems, Madison, WI.

[v] SHELXTL v2008/4, © 2008, Bruker Analytical X-ray Systems, Madison, WI.

Table S2: Crystallographic data

Code	**14XB:E**	**135XB:E**	**12XB:B**	**135XB:A**
Formula moiety	$C_{39}H_{42}N_6$, $C_6F_4I_2$	$C_{39}H_{42}N_6$, $C_6F_3I_3$, $C_4H_8O_2$	$C_{21}H_{24}N_6$, $C_6F_4I_2$	$C_{21}H_{24}N_6$, $C_6F_3I_3$
Empirical formula	$C_{45}H_{42}F_4I_2N_6$	$C_{49}H_{50}F_3I_3N_6O_2$	$C_{27}H_{24}F_4I_2N_6$	$C_{27}H_{24}F_3I_3N_6$
Molecular weight	996.64	1192.65	762.32	870.22
Color, Habit	Colorless, Prism	Colorless, Plates	Colorless, Plates	Colorless, Prism
Crystal system	Monoclinic	Triclinic	Triclinic	Orthorhombic
Space group, Z	$P2(1)/c$, 4	$P\bar{1}$, 2	$P\bar{1}$, 2	$Pbca$, 8
a, Å	16.337(6)	9.764(4)	9.255(3)	7.931(2)
b, Å	16.340(5)	11.594(4)	11.995(4)	20.310(5)
c, Å	15.642(5)	22.901(9)	13.507(5)	37.342(10)
α, º	90	101.90(2)	78.82(2)	90
β, º	102.862(13)	97.56(3)	84.15(2)	90
γ, º	90	99.98(2)	68.959(19)	90
Volume, Å^3	4071(2)	2460.4(16)	1372.1(8)	6015(3)
Density, g/cm^3	1.626	1.610	1.845	1.922
T, ºK	130(2)	180(2)	200(2)	296(2)
Crystal size, min x mid x max	0.164 x 0.182 x 0.284	0.097 x 0.154 x 0.208	0.078 x 0.124 x 0.268	0.204 x 0.268 x 0.294
X-ray wavelength, Å	0.71073	0.71073	0.71073	0.71073
μ, mm^{-1}	1.604	1.961	2.348	3.164
Trans min / max	0.66 / 0.78	0.69 / 0.83	0.57 / 0.84	0.46 / 0.56
θ_{min}, º	1.28	0.92	1.54	1.09
θ_{max}, º	25.68	25.96	25.93	25.71
Reflections				
collected	56460	62625	27366	115218

independent	7640	8908	5263	5700
observed	5489	7102	3485	4007
R_{int}	0.0838	0.0681	0.1018	0.0717
Threshold expression	> 2$\sigma(I)$	> 2$\sigma(I)$	> 2$\sigma(I)$	> 2$\sigma(I)$
No. parameters	523	579	356	356
No. restraints	0	0	0	0
R_1 (observed)	0.0442	0.0338	0.0497	0.0638
wR_2 (all)	0.1198	0.1100	0.1773	0.1740
Goodness of fit (all)	1.101	1.046	1.050	1.243
ρ_{max}, ρ_{min}, e Å^{-3}	0.688, -1.023	0.629, -0.983	1.112, -1.249	1.046, -0.909
Completeness to 2θ limit	0.988	0.927	0.982	0.994

4. Melting Points

Table S3: Melting points

Co-crystal	Melting points (°C)		
	Acceptor	Donor	Co-crystal
12XB:B	133	49-50	98-99
135XB:A	214-215	152	177-178
135XB:E	285-290	152	219
14XB:E	285-290	108-110	229
